# Wells syndrome: emerging triggers and treatments– an updated systematic review

**DOI:** 10.1007/s00403-025-04305-9

**Published:** 2025-06-09

**Authors:** Areeba Ahmed, Brian Cahn, Roger Haber

**Affiliations:** 1 Department of Dermatology, Northwestern University Feinberg School of Medicine, Chicago, IL USA; 2 Department of Dermatology, Columbia University, New York, NY USA; 3 Department of Dermatology, University of Illinois Chicago, Chicago, IL USA

**Keywords:** Wells syndrome, Eosinophilic cellulitis, COVID-19, Vaccination, Biologic therapy, Eosinophilic dermatosis

## Abstract

Importance: Wells syndrome (eosinophilic cellulitis) is a rare inflammatory dermatosis characterized by erythematous, edematous plaques and dermal eosinophilic infiltration. Understanding its evolving triggers and treatment options is critical for optimizing management, particularly in corticosteroid-refractory cases. Objective: To systematically review newly reported immunologic and iatrogenic triggers of Wells syndrome, as well as emerging therapies, with the goal of updating clinical guidance. This review focuses on diagnosis and therapy, emphasizing outcomes in patients with refractory or relapsing disease. Evidence review: A systematic literature search was conducted following PRISMA 2020 guidelines across six databases for English-language studies published between January 2016 and May 2025. Studies were eligible if they described new triggers or treatments for Wells syndrome. Article selection and data extraction were performed independently by two reviewers. Risk of bias was assessed using the Joanna Briggs Institute and Newcastle-Ottawa tools. Findings: Twenty-four studies met inclusion criteria: 21 case reports, 2 case series, and 1 retrospective cohort study. Newly identified triggers included COVID-19 infection, SARS-CoV-2 and influenza vaccines, aluminum- and gelatin-containing pediatric vaccines, and biologic therapies such as ustekinumab and tumor necrosis factor-alpha (TNF-α) inhibitors. In vaccine-related cases, causality was supported by positive patch testing. Novel therapies trialed in corticosteroid-refractory or relapsing patients included dupilumab, topical ruxolitinib, abrocitinib, and mepolizumab. Most patients experienced complete or near-complete resolution. However, recurrences were common, particularly in idiopathic cases or upon re-exposure to known triggers. Conclusions and relevance: Recent literature expands the clinical spectrum of Wells syndrome, highlighting new immunologic and iatrogenic triggers. Targeted treatments, especially biologics and Janus kinase inhibitors, demonstrate promising results and may offer steroid-sparing alternatives for patients with refractory disease. Clinicians should consider emerging triggers in differential diagnosis and evaluate newer therapies in recurrent or treatment-resistant cases. Further prospective and registry-based studies are warranted to validate efficacy and support development of evidence-based management guidelines.

**Supplementary Information:**

The online version contains supplementary material available at 10.1007/s00403-025-04305-9.

## Introduction

### Overview of wells syndrome

Wells syndrome is a rare, relapsing dermatosis marked by eosinophilic plaques and distinctive flame figures. Often mistaken for infection or autoimmune disease, it typically resolves on its own but may recur without proper trigger identification and management [1]. 

It typically presents with pruritic, erythematous plaques, often on the limbs or trunk. Some cases develop bullae or nodules, mimicking infection or vasculitis. Histology shows eosinophil-rich infiltrates and flame figures, though these are not specific and may overlap with other dermatoses [2, 3]. It usually resolves within weeks but often recurs. Peripheral eosinophilia occurs in about half of cases but lacks diagnostic reliability. Its variable presentation can delay recognition and treatment [4]. It is thought to result from a type IV hypersensitivity reaction, with eosinophil activation causing dermal inflammation and flame figures. Triggers include insect bites, infections, medications, malignancies, and eosinophilic systemic diseases [5, 6]. 

A 2016 review of 126 cases found most Wells syndrome cases were idiopathic, with no clear trigger. Clinical and treatment variability limited standardized management [7]. Pre-2016 literature was largely limited to case reports and small series, lacking standardized diagnostic criteria or consistent treatment approaches. Corticosteroids were commonly used but often led to relapse. Long-term outcomes for alternative therapies were poorly documented. Recent reports suggest new triggers and treatments, justifying updated evaluation.

COVID-19 has emerged as a potential trigger for Wells syndrome. A 2022 case showed Wells as the initial skin manifestation of SARS-CoV-2, with biopsy confirming eosinophilic infiltration. The eruption resolved with corticosteroids [8]. Like other viral agents, COVID-19 may induce eosinophilic dermatoses via delayed hypersensitivity mechanisms [1]. Post-vaccination Wells syndrome has been reported in both children and adults since 2016, following vaccines like DTaP-IPV, MMR, pneumococcal, and influenza. Patch testing in several cases confirmed hypersensitivity to excipients such as aluminum, gelatin, thimerosal, and neomycin [9, 10]. Recent reports link COVID-19 vaccines to Wells syndrome, with excipients like polyethylene glycol suspected as triggers. Affected patients typically recovered with corticosteroids or antihistamines. These findings underscore the need to review vaccination history and excipients in eosinophilic skin eruptions [11]. Though rare, Wells syndrome has been reported as a reaction to biologics, including TNF-α inhibitors and IL-12/23 blockers [12]. Cases with adalimumab and ustekinumab showed symptom resolution after stopping the drug. These therapies may alter cytokine balance, triggering eosinophilic inflammation in susceptible patients [13, 14]. 

Although effective, long-term corticosteroid use in Wells syndrome is limited by systemic side effects. Recent case reports support alternatives including dupilumab, mepolizumab, and JAK inhibitors in refractory disease. Dupilumab led to sustained remission even after a brief 4-dose course [15, 16]. Mepolizumab was successful in patients with severe or overlapping eosinophilic conditions [17]. Topical ruxolitinib and oral abrocitinib showed efficacy in localized and steroid-resistant cases, highlighting the role of cytokine-targeted therapies [18, 19]. 

### Rationale and objectives

Wells syndrome is often underdiagnosed due to its rarity and resemblance to other dermatoses. Before 2016, evidence was limited to case reports without standardized criteria or consistent outcomes. Since then, new associations with COVID-19, vaccines, and biologic therapies have emerged, alongside the use of novel treatments like dupilumab, mepolizumab, and JAK inhibitors. However, their overall impact has not been systematically reviewed. This evolving clinical landscape warrants synthesis of recent data to clarify triggers, support differential diagnosis, and evaluate the effectiveness of these emerging therapies in guiding safer, more individualized care.

The objective of this review is to summarize all published evidence from 2016 to 2025 on emerging triggers and treatment strategies for Wells syndrome. It aims to identify novel associations such as infections, vaccinations, and biologics, describe common clinical and histopathologic features, assess therapeutic outcomes with agents like dupilumab, mepolizumab, and JAK inhibitors, examine recurrence and diagnostic trends, and highlight gaps in the current evidence base to guide future research.

## Methods

### Review design and protocol registration

This review followed the PRISMA 2020 guidelines, which provide a structured method for reporting systematic reviews to ensure transparency and reproducibility across study selection, analysis, and synthesis processes [20]. A PRISMA flow diagram was used to document each review stage, and any protocol deviations were justified per Cochrane recommendations [21]. 

An internal protocol was created and peer-reviewed before the search, covering objectives, criteria, strategy, and bias assessment. Due to the narrow scope and time constraints, the review was not registered with PROSPERO, though the protocol was archived. Future updates may pursue registration.

As the study relied solely on published, de-identified data, it was exempt from institutional ethical review [22]. Standard ethical practices, including citation accuracy, data integrity, and conflict-of-interest transparency, were strictly followed.

### Eligibility criteria

Eligibility was based on the PICOS framework [9]. Included were patients of any age with a clinical and histopathologic diagnosis of Wells syndrome, regardless of peripheral eosinophilia or trigger identification. *Studies on exposures* included newly recognized triggers reported since 2016, such as COVID-19, vaccinations, and biologics. Treatments of interest involved any therapy beyond conventional corticosteroids, particularly dupilumab, anti–IL-5 agents, and JAK inhibitors.

No comparator was required, as most reports were observational. Outcomes assessed included lesion resolution, time to improvement, recurrence, and adverse effects. Trigger-related outcomes included temporal associations or positive patch testing [9]. 

Eligible study types were case reports, series, cohort, and cross-sectional studies. Reviews, abstracts, commentaries without original cases, non-English papers, laboratory-only studies, and animal models were excluded.

### Information sources and search strategy

We conducted a systematic search of PubMed, Embase, Scopus, Web of Science, Cochrane Library, and Google Scholar to identify relevant literature on Wells syndrome. Reference lists of included articles were also manually screened to capture additional studies.

Search terms combined free text and MeSH headings using Boolean logic. Key terms included “Wells syndrome,” “eosinophilic cellulitis,” “COVID-19,” “vaccination,” “biologic therapy,” “dupilumab,” “mepolizumab,” and “JAK inhibitor.” The complete search strategy is presented in Table 1.

**Table 1:** Characteristics of included studies

Study (Year)	Design	Patients (*n*)	New Trigger(s) Described	Novel Treatment(s) Described
Yu et al. (2018) [9]	Case report	1 (4-year-old boy)	Vaccination (multiple pediatric vaccines)– confirmed by patch-test to neomycin & aluminum	– (Topical steroids; trigger avoidance)
Fournier et al. (2020) [10]	Case series	2 (children)	Vaccination (various pediatric vaccines)– patch-test positive to aluminum salts	– (Topical/systemic steroids)
Granja et al. (2025) [26]	Case report	1 (12-month boy)	Vaccination (MMR, pneumococcal, meningococcal)– patch-test positive to gelatin	– (Systemic steroids)
Praturlon et al. (2025) [35]	Case report	1 (child)	Vaccination (not specified, recurrent episodes post-vaccination)	– (Systemic steroids)
Moseley et al. (2022) [8]	Case report	1 (51 F)	COVID-19 infection (Wells as presenting sign of COVID-19)	– (Systemic steroids)
Ikediobi et al. (2022) [11]	Case report	1 (12 M)	COVID-19 mRNA vaccine (Pfizer)– likely triggered by polyethylene glycol excipient	– (Topical steroids, antihistamines)
Šajn et al. (2022) [36]	Case report	1 (46 F)	Hematologic malignancy (CLL) and influenza vaccination (thimerosal) and ibrutinib– most likely trigger: thimerosal vaccine	– (Oral steroids)
Dabas et al. (2018) [12]	Case report + review	1 (36 M)	Biologic (adalimumab biosimilar for HS)– Wells induced after 6 months of therapy	– (Drug stopped; oral steroids)
Rozenblat et al. (2019) [14]	Case report	1 (58 M)	Biologic (ustekinumab for psoriasis)– Bullous Wells induced after multiple doses	– (Drug stopped; steroids)
Kim et al. (2023) [37]	Case report	1 (64 M)	Biologic (ustekinumab for psoriasis)– recurrent Wells flares after each injection	– (Drug stopped; steroids)
Heinig et al. (2019) [5]	Case series/review	4 (adults)	Various (1 with unknown trigger, 1 drug-induced, etc.)– overview	– (Conventional treatments)
**Novel treatment-focused**				
Heelan et al. (2018) [29]	Case report	1 (adult)	– (Idiopathic WS with asthma)	Mepolizumab (anti–IL-5)– successful remission
Herout et al. (2018) [38]	Case report	1 (adult)	– (Wells with asthma)	Mepolizumab– successful (*same case as above*)
McMullan et al. (2021) [39]	Case report	1 (adult)	– (Idiopathic)	Dupilumab– successful (refractory case)
Kirven & Plotner (2023) [15]	Case report	1 (adult)	– (Idiopathic)	Dupilumab– short 4-dose course, successful
McMullan et al. (2023) [39]	Case report	1 (85 F)	– (Atypical neutrophil-rich variant)	Dupilumab + steroids– successful
Monroe et al. (2024) [19]	Case report	1 (58 F)	– (Localized recurrent WS)	Topical ruxolitinib– successful, prevented flares
Cheng et al. (2025) (early view) [18]	Case report	1 (adult)	–	Abrocitinib (oral JAK1)– successful (refractory case)
Tirado-Sánchez et al. (2021) [6]	Retro. series	35 (adults)	Various (in 40%: insect bites; 11% drug; 9% vaccine)	Conventional (steroids in 90%; dapsone 15%; etc.)
Terhorst-Molawi et al. (2020) [32]	Case report	1 (adult)	– (Refractory idiopathic Wells syndrome)	Mepolizumab– effective in steroid-resistant case
Iglesias Puzas et al. (2017) [40]	Case report	1 (adult)	– (Recurrent idiopathic Wells syndrome)	Colchicine– used as steroid-sparing agent with good response
Larangeira de Almeida Junior et al. (2025) [31]	Case report	1 (adult)	– (Idiopathic)	Dupilumab– short course (4 weeks), complete resolution
Su et al. (2025) [41]	Case report	1 (adult)	– (Steroid-refractory case)	Abrocitinib (oral JAK1 inhibitor)– successful remission
Shah et al. (2023) [4]	Case report + review	1 (adult)	–	Dupilumab successful; literature review of 4 cases

No restrictions were applied to study design or publication status, though only English-language, human studies were included. Duplicates were removed using EndNote X20, and screening was performed independently by two reviewers, with disagreements resolved by a third.

The search covered literature from January 1, 2016, to May 1, 2025. Older studies were reviewed for context only. A final update was conducted just before data extraction to ensure the most current literature was included.

### Selection process

**Fig. 1:**
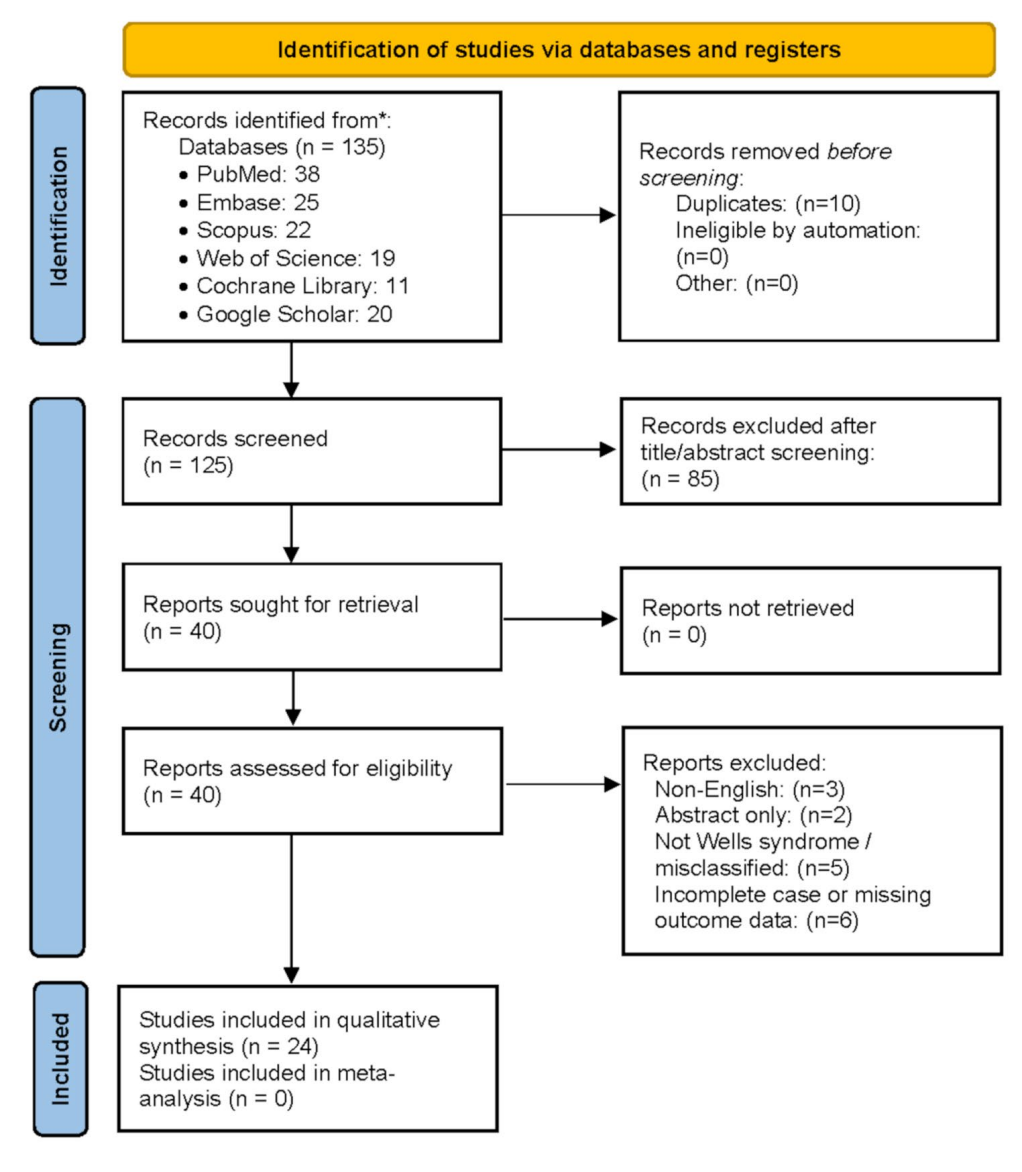
PRISMA 2020 flow diagram of study selection (2016–2025)

All records were imported into a reference manager, and duplicates were removed. Two reviewers (AA and BC) independently screened titles and abstracts, followed by full-text review. Disagreements were resolved by discussion or third-party adjudication (RH). The PRISMA flow diagram (Fig. 1) summarizes the selection process [22]. Full-text exclusions and reasons are detailed in the appendix.

### Data collection process

We used a standardized form to extract data on patient characteristics, triggers, diagnostics, treatment, outcomes, and follow-up. For observational studies, we recorded setting, sample size, and aggregate results. One reviewer extracted data, and a second verified accuracy. Ambiguities were resolved through detailed text review; authors were not contacted.

### Data items and outcomes

Key variables were predefined. We noted if studies applied diagnostic criteria such as those by Heelan et al. and recorded relevant findings, including eosinophilia, flame figures, and immunofluorescence results [3]. Clinical data included lesion type, distribution, systemic symptoms, and whether the case was new or relapsing. We documented potential triggers, including infections, vaccines, biologics, and underlying conditions.

Treatments were categorized as conventional or novel (e.g., dupilumab, JAK inhibitors), with dosing noted when available. Outcomes included response, time to improvement, recurrence, and adverse events. For trigger-related cases, patch test results were also recorded [9]. Follow-up duration and quality indicators, such as alternative diagnoses being ruled out, were also extracted.

### Risk of bias assessment

We used Joanna Briggs Institute (JBI) checklists for case reports and case series to assess methodological quality [23]. Two reviewers independently evaluated items such as diagnostic clarity, intervention details, and outcomes. The single retrospective study was appraised using the Newcastle-Ottawa Scale (NOS) for observational studies [24]. Discrepancies were resolved by consensus. Although no studies were excluded for quality concerns, bias assessments informed evidence interpretation. Funnel plots were not applicable due to the narrative nature of the data.

### Synthesis of results

Due to heterogeneity and descriptive data, we performed a qualitative synthesis. Studies were grouped by triggers and treatments, and outcomes were compared by timing, frequency, and response durability, especially for dupilumab and JAK inhibitors. No meta-analysis was done. Findings are summarized in tables and interpreted alongside pre-2016 literature to highlight recent developments.

## Results

### Study selection

After deduplication, 125 records were screened, with 5 more identified manually. Of 40 full-texts assessed, 15 were excluded for language, format, misclassification, or insufficient data. We included 24 studies: 21 case reports, 2 case series, and 1 retrospective cohort. No interventional trials were found. The PRISMA diagram (Fig. 1) summarizes the selection process. Table 2 outlines study characteristics, which span all age groups and cover both triggers and treatment responses, with some overlap between categories.

**Table 2:** Summary of emerging triggers and therapeutic strategies reported in wells syndrome (2016–2025)

**Emerging Triggers**	**No. of cases**	**Comments/Outcomes**
COVID-19 infection	1–2	Wells syndrome can present with or shortly follow COVID-19 [41]. Resolved with steroids; no long-term sequelae.
Vaccinations (general)	∼ 9	Includes pediatric vaccines (DTaP, MMR, etc.) [9], influenza [28], COVID-19 vaccines [11]. Onset 1–14 days post-vaccination. All patients recovered (most with steroids). Specific antigens identified: thimerosal [28], aluminium, gelatin [10], PEG [11]. Avoidance prevents recurrence.
Pediatric immunizations	4 (3 reports)	Aluminium-adjuvanted vaccines triggering Wells in children; confirmed by patch tests. Good prognosis [9].
Influenza vaccine	1	Adult with CLL on ibrutinib; likely thimerosal reaction. Resolved with steroids, no change in CLL therapy [28].
COVID-19 vaccines	3–4	Both mRNA (Pfizer) and viral vector (AstraZeneca) reported. Likely PEG or polysorbate allergy. Lesions responsive to steroids; no recurrence noted on avoiding 2nd dose or premedication [11].
Biologic therapies (drug-induced Wells)	∼ 5	Ustekinumab (2 cases), adalimumab (1), infliximab (prior lit), possibly ibrutinib (1, confounded). All required drug cessation + steroids; no further flares after discontinuation ^12,25^.
Others: New drug triggers	1–2	Thiazide diuretic (1 case)– uncommon. Emphasizes any new med could be suspect in idiopathic cases [29].
Underlying disease associations	2	CLL/NHL (2 cases)– aligns with known malignancy associations. Treating the malignancy or Wells as needed; outcomes good [28].
**Novel Treatments**	**No. of cases**	**Comments/Outcomes**
Dupilumab (IL-4/IL-13 inhibitor)	5+	High success in steroid-refractory Wells. All cases showed clearance or marked improvement, with no relapses during therapy (and none in one case after stopping). Well tolerated. Suggests a steroid-sparing maintenance option. ^31,39^
Anti-IL-5 (Mepolizumab)	2–3	Resolved Wells in eosinophilic asthma patients. One with EGPA overlap improved completely. Supports targeting eosinophils; use case likely when concomitant hypereosinophilic conditions exist. [38]
Anti-IgE (Omalizumab)	1	One reported success (prior to 2016, plus noted in 2025 summary). Might help in atopic-associated Wells, but minimal data. [31]
JAK inhibitors (systemic)	1–2	Abrocitinib (JAK1) case(s) with complete remission. Implies JAK-STAT pathway importance. More data needed; consider for severe cases if other biologics fail. [30]
JAK inhibitors (topical)	1	Ruxolitinib cream for localized Wells– rapid control of flares. Offers a local therapy option for limited disease; no systemic exposure. [19]
Other therapies (re-evaluated)	–	Colchicine: evidence of benefit in recurrent Wells (1 case, 2017)– decreased flares. Dapsone, methotrexate, cyclosporine: sporadic use continued in some series with partial success. Steroids remain first-line for acute flares [11, 31, 40].

### Quality appraisal

The evidence was low quality, mainly case reports and small series. While most met JBI criteria and confirmed diagnosis by biopsy, about 30% lacked adequate differential assessment, increasing bias risk. Case series like Fournier et al. used stronger methods, including patch testing [10]. The retrospective study scored moderately on NOS but lacked controls. COVID-linked and rare cases may be overrepresented due to publication bias. Findings were consistent but warrant cautious interpretation.

### Clinical features and diagnosis of wells syndrome in included studies

Most patients showed classic features; erythematous, edematous plaques often mistaken for cellulitis or urticaria. Bullous forms were reported, including with ustekinumab and during COVID-19 [8, 25]. A neutrophil-rich scalp variant was seen in an older adult [2]. Pediatric cases often involved localized or vaccine-related lesions [9, 10, 26]. 

Histology confirmed eosinophilic infiltrates and flame figures in nearly all cases. One COVID-linked case showed deeper panniculitis [27]. Direct immunofluorescence was negative where tested. Peripheral eosinophilia was present in about half of adults but not essential for diagnosis. Diagnostic criteria proposed by Heelan et al. were applied in some reports [3]. Moseley et al. identified a case meeting three major and one minor criterion, reinforcing diagnostic consistency across studies [8]. Overall, while symptom patterns remained typical, these cases highlight evolving triggers and management approaches.

### New triggers associated with wells syndrome (2016–2025)

Recent reports broaden the known triggers of Wells syndrome to include viral infections, vaccines, biologics, and other immunologic exposures.

#### COVID-19 infection

SARS-CoV-2 has been linked to Wells syndrome, including a case with eosinophilic lesions confirmed by biopsy and positive COVID-19 PCR shortly after respiratory symptoms [8]. Another case involved eosinophilic panniculitis, possibly a deeper variant of the disease [1]. These suggest that COVID-19 may trigger Wells via hypersensitivity pathways.

#### Vaccinations

Wells syndrome has followed several vaccines since 2016. Pediatric cases linked to DTaP-IPV, MMR, and pneumococcal vaccines showed sensitization to excipients like aluminum and gelatin [9, 10]. One case implicated thimerosal in influenza vaccine-associated Wells in a patient on ibrutinib [28]. COVID-19 vaccines have also been involved, with PEG and polysorbate 80 identified as probable antigens. Most patients recovered with corticosteroids or antihistamines [11]. Onset typically occurred within 1–14 days, and patch testing confirmed excipient sensitivity in several cases.

#### Biologic and immunomodulatory therapies

Several biologics have been associated with Wells syndrome. Ustekinumab triggered recurrent or bullous lesions in two patients, both of whom improved after stopping the drug [14, 25]. TNF-α inhibitors such as adalimumab and infliximab have also been linked, likely due to altered cytokine balance [28]. Targeted cancer therapies like ibrutinib were suspected in one case, though confounded by concurrent vaccination. No cases were associated with checkpoint inhibitors [28]. 

#### Other medications and conditions

A thiazide diuretic was proposed as a trigger in one patient with eczema [29]. No new antibiotic-related cases were found, though penicillin has been implicated historically. Hematologic malignancies remain relevant; Wells syndrome occasionally preceded or accompanied diagnoses such as CLL or lymphoma [28]. 

### Novel treatments and management strategies

Corticosteroids remain first-line for Wells syndrome, but concerns over relapse and adverse effects have prompted interest in steroid-sparing therapies. Since 2016, case reports highlight promising outcomes with newer biologics and JAK inhibitors.

#### Dupilumab (IL-4/IL-13 pathway blockade)

As an IL-4 receptor antagonist, dupilumab has shown efficacy in several cases. Patients with refractory or relapsing Wells syndrome achieved remission, including one after just four doses [15, 16]. It was also effective in a neutrophil-rich variant [2]. Responses were rapid and sustained, with minimal side effects, supporting its role as a disease-modifying option.

#### Anti–IL-5 therapy (mepolizumab)

Mepolizumab, targeting eosinophils, was successful in patients with Wells syndrome and eosinophilic comorbidities. Two reports showed complete remission without corticosteroids [3, 17]. Though data are limited, the biologic rationale and clinical outcomes are encouraging.

#### Janus kinase inhibitors

JAK inhibitors have emerged as effective options in select cases. Topical ruxolitinib controlled localized, recurrent lesions [19], while oral abrocitinib achieved remission in a steroid-resistant case [18]. These agents offer targeted cytokine suppression, especially useful in patients unsuitable for systemic steroids.

#### Other therapies

Colchicine, dapsone, and methotrexate showed variable success, whereas targeted therapies like dupilumab, mepolizumab, and JAK inhibitors offered more consistent control and fewer relapses, with minimal reported side effects despite limited follow-up. A comparative summary of treatment responses, durability, and safety across these agents is provided in Table 3.

**Table 3:** Complete search strategy used for the systematic review

Database	Search Terms Used
**PubMed/MEDLINE**	(“Wells syndrome“[Title/Abstract] OR “eosinophilic cellulitis“[Title/Abstract]) AND (“case report“[Publication Type] OR “case series“[Title/Abstract] OR “observational study“[Title/Abstract] OR “clinical trial“[Title/Abstract]) AND (“COVID-19” OR “vaccination” OR “biologic” OR “dupilumab” OR “mepolizumab” OR “JAK inhibitor”)
**Embase**	‘wells syndrome’/exp OR ‘eosinophilic cellulitis’/exp AND (‘case report’/exp OR ‘case series’/exp OR ‘observational study’/exp OR ‘clinical trial’/exp) AND (‘covid-19’/exp OR ‘vaccination’/exp OR ‘biologic’/exp OR ‘dupilumab’/exp OR ‘mepolizumab’/exp OR ‘jak inhibitor’/exp)
**Scopus**	TITLE-ABS-KEY(“Wells syndrome” OR “eosinophilic cellulitis”) AND TITLE-ABS-KEY(“case report” OR “case series” OR “observational study” OR “clinical trial”) AND TITLE-ABS-KEY(“COVID-19” OR “vaccination” OR “biologic” OR “dupilumab” OR “mepolizumab” OR “JAK inhibitor”)
**Web of Science**	TS=(“Wells syndrome” OR “eosinophilic cellulitis”) AND TS=(“case report” OR “case series” OR “observational study” OR “clinical trial”) AND TS=(“COVID-19” OR “vaccination” OR “biologic” OR “dupilumab” OR “mepolizumab” OR “JAK inhibitor”)
**Cochrane Library**	(“Wells syndrome” OR “eosinophilic cellulitis”) in Title/Abstract/Keywords AND (“COVID-19” OR “vaccination” OR “biologic” OR “dupilumab” OR “mepolizumab” OR “JAK inhibitor”) in Title/Abstract/Keywords
**Google Scholar**	“Wells syndrome” OR “eosinophilic cellulitis” AND (“case report” OR “COVID-19” OR “vaccination” OR “biologic” OR “dupilumab” OR “mepolizumab” OR “JAK inhibitor”)

### Outcomes and recurrence

Wells syndrome generally follows a benign course, with most cases resolving with corticosteroids. Recent reports from 2016 to 2025 show rapid improvement, often within days to weeks, particularly when eosinophilic inflammation is prominent. Topical treatment sufficed in milder or vaccine-linked cases [9–11]. Recurrence was uncommon in treatment-naïve patients when triggers were removed.

However, relapse remains frequent, especially in idiopathic or chronic cases. Over half of patients in a retrospective study experienced recurrence [6]. Ustekinumab-associated Wells syndrome often flared after each dose [14, 25]. Avoiding re-exposure—whether to vaccines or biologics—often prevented further episodes [12, 14, 25]. 

Targeted therapies showed durable control. Dupilumab prevented recurrence even after a brief course [15], and mepolizumab was similarly effective in eosinophil-driven disease [17]. Topical ruxolitinib offered reliable, localized flare suppression, supporting individualized, steroid-sparing strategies [19]. 

Prognosis was favorable, with no severe complications reported. Biologics were well tolerated, and outcomes were better than with traditional immunosuppressants. Key findings are summarized in Table 4.

**Table 4:** Comparative efficacy and durability of biologic and JAK inhibitor therapies in wells syndrome (2016–2025)

Therapy	Study (Year)	Response Time	Remission Duration	Relapse Reported	Adverse Events
**Dupilumab**	Traidl et al. (2021) [16]	2–3 weeks	Sustained on therapy	No	None
	Kirven & Plotner (2023) [15]	Within 3 weeks	10 months (4 doses only)	No	None
	McMullan et al. (2023) [2]	Few weeks	Maintained during treatment	No	None
	Larangeira de Almeida (2025) [31]	Within 1 month	Remission at 4-week mark	No	None
	Shah et al. (2023) [4]	Not reported	Sustained (literature-based)	No	None
**Mepolizumab**	Herout et al. (2018) [17]	Few weeks	Sustained on therapy	No	None
	Terhorst-Molawi et al. (2020) [32]	4–6 weeks	Sustained on therapy	No	None
**Abrocitinib**	Su et al. (2025) [18]	1–2 weeks	Maintained through follow-up	No	None
**Ruxolitinib (topical)**	Monroe et al. (2024) [19]	Within days	Controlled with reapplication	No	None
**Colchicine**	Iglesias Puzas et al. (2017) [40]	Not specified	Long-term flare control	No	None

## Discussion

This review highlights evolving triggers and treatments in Wells syndrome, showing how even rare conditions are shaped by global events and therapeutic progress.

### Emerging triggers

Associations between Wells syndrome and COVID-19 or vaccination are rare but increasingly reported. While incidence remains low, repeated global observations suggest a real link [8, 11]. These cases highlight Wells syndrome as an immune-mediated reaction, potentially part of the broader spectrum of COVID-related dermatoses [11]. Recognizing temporal links to vaccination can prevent misdiagnosis and guide patch testing, particularly in pediatric patients [9].

Similarly, biologics like ustekinumab and TNF inhibitors may provoke Wells syndrome as part of known paradoxical reactions. Though rare, clinicians should consider these drugs when eosinophilic rashes arise during treatment [30]. 

### Diagnostic criteria

Recent use of formal diagnostic criteria marks progress toward standardizing Wells syndrome diagnosis. The proposed system—major features like clinical and histologic findings, plus minor criteria such as eosinophilia or trigger, captured nearly all cases in this review [8]. Adoption of a uniform case definition could support future registries or trials.

### Implications of new treatments

Targeted therapies like dupilumab and mepolizumab represent a shift in Wells syndrome management, offering effective, steroid-sparing alternatives for relapsing cases [17, 31]. These agents may be considered earlier in patients with comorbid allergic conditions. Their use also reflects a precision medicine approach, mepolizumab for eosinophilic asthma and dupilumab for Th2-driven disease [2, 17]. Their success supports IL-4/13 and IL-5 as key pathways, reinforcing both clinical and mechanistic rationale [2, 32]. 

### Quality of evidence

Evidence remains limited to case reports and small series, which are prone to bias—positive outcomes are more likely to be published. Failures, such as non-response to dupilumab, may go unreported, skewing perceptions of efficacy. Short follow-up also limits long-term conclusions. Without control groups, causal links to triggers like vaccines are difficult to confirm and rely on author judgment and patch testing [11]. The lone retrospective study added some context but lacked detail. This review aimed to document emerging patterns, not estimate incidence.

### Clinical recommendations vs. evidence

Due to limited evidence, recommendations, such as using dupilumab, remain provisional. A prospective series or small trial in chronic Wells patients could clarify efficacy, especially given dupilumab’s availability in atopic care. A disease registry could also help validate findings. Given the rarity of Wells syndrome, international collaboration will be key to advancing research.

### Comparison with 2016 review

The 2016 review by Rässler et al. outlined treatments through 2015 but lacked trial data and emphasized corticosteroids [7]. Our 2016–2025 update highlights anecdotal success with targeted therapies like dupilumab, anti–IL-5 agents, and JAK inhibitors. Unlike earlier work, we also identify new triggers such as vaccines and COVID-19, reflecting evolving clinical realities.

### Limitations of our review process

Despite a broad search, some cases may have been missed, especially non-English reports—one French study of 11 pediatric cases was excluded [33]. We did not conduct a meta-analysis due to heterogeneity, so no pooled estimates were generated. NOS was applied qualitatively to describe bias, though it has limitations for case series.

### Future directions

Emerging therapies suggest several research priorities. The effectiveness of IL-4/13 and IL-5 inhibitors warrants further investigation into cytokine profiles in Wells lesions, especially given evidence of JAK-STAT pathway activation [34]. Genetic predisposition, such as HLA types or atopic backgrounds, may explain individual susceptibility to vaccine-triggered cases but remains unstudied. Preventative strategies like antihistamine premedication have been proposed for vaccine-related Wells, particularly in children, though formal guidelines are lacking and current practice varies [11]. Controlled trials of dupilumab or mepolizumab could clarify efficacy but would require multicenter collaboration due to case rarity. Lastly, long-term outcome data are limited. It remains unclear whether remission persists after therapy discontinuation, making extended follow-up essential.

## Conclusion

Wells syndrome is a rare eosinophilic dermatosis increasingly linked to infections, vaccines, and biologics. While corticosteroids remain standard, targeted therapies like dupilumab, mepolizumab, and JAK inhibitors show promise in resistant cases. This review highlights post-2016 trends in triggers and treatment, with most patients improving through trigger avoidance or biologic use. Though findings are observational, they underscore the need for early recognition and personalized management. Continued reporting and long-term studies are vital to guide evidence-based care.

## Electronic supplementary material

Below is the link to the electronic supplementary material.

Supplementary Material 1

## Data Availability

No datasets were generated or analysed during the current study.
