# Supplementary material for: Wells syndrome: emerging triggers and treatments– an updated systematic review
Source: Arch Dermatol Res. 2025 Jun 9;317(1):805. doi: 10.1007/s00403-025-04305-9 (PMC12149015; doi:10.1007/s00403-025-04305-9)
Supplement: Supplementary file 1 — Supplementary Material 1 [file 403_2025_4305_MOESM1_ESM.docx]

**Supplementary Table 1.** JBI Critical Appraisal Checklist for Case Reports (n = 21)

| **Study (Year)** | **Clear Patient History** | **Diagnostic Clarity** | **Intervention Described** | **Post-Intervention Outcome** | **Alternative Diagnoses Considered** | **Follow-up Described** | **Quality Rating** |
| --- | --- | --- | --- | --- | --- | --- | --- |
| Yu et al. (2018) ^1^ | Yes | Yes | Yes | Yes | Yes | No | Moderate |
| Granja et al. (2025) ^2^ | Yes | Yes | Yes | Yes | Yes | Yes | High |
| Praturlon et al. (2025) ^3^ | Yes | Yes | Yes | Yes | No | Yes | Moderate |
| Moseley et al. (2022) ^4^ | Yes | Yes | Yes | Yes | Yes | No | Moderate |
| Ikediobi et al. (2022) ^5^ | Yes | Yes | Yes | Yes | Yes | No | Moderate |
| Šajn et al. (2022) ^6^ | Yes | Yes | Yes | Yes | Yes | No | Moderate |
| Dabas et al. (2018) ^7^ | Yes | Yes | Yes | Yes | Yes | Yes | High |
| Rozenblat et al. (2019) ^8^ | Yes | Yes | Yes | Yes | Yes | No | Moderate |
| Kim et al. (2023) ^9^ | Yes | Yes | Yes | Yes | Yes | No | Moderate |
| Heelan et al. (2018) ^10^ | Yes | Yes | Yes | Yes | Yes | Yes | High |
| Herout et al. (2018) ^11^ | Yes | Yes | Yes | Yes | Yes | Yes | High |
| McMullan et al. (2023) ^12^ | Yes | Yes | Yes | Yes | No | Yes | Moderate |
| Kirven & Plotner (2023) ^13^ | Yes | Yes | Yes | Yes | Yes | No | Moderate |
| McMullan et al. (2023) ^14^ | Yes | Yes | Yes | Yes | No | Yes | Moderate |
| Monroe et al. (2025) ^15^ | Yes | Yes | Yes | Yes | Yes | Yes | High |
| Cheng et al. (2025) ^16^ | Yes | Yes | Yes | Yes | Yes | No | Moderate |
| Terhorst-Molawi et al. (2020) ^17^ | Yes | Yes | Yes | Yes | Yes | Yes | High |
| Iglesias Puzas et al. (2017) ^18^ | Yes | Yes | Yes | Yes | Yes | No | Moderate |
| Larangeira de Almeida (2025) ^19^ | Yes | Yes | Yes | Yes | Yes | No | Moderate |
| Su et al. (2025) ^16^ | Yes | Yes | Yes | Yes | Yes | Yes | High |
| Shah et al. (2023) ^20^ | Yes | Yes | Yes | Yes | Yes | No | Moderate |

**Supplementary Table 2. JBI Critical Appraisal Checklist for Case Series (n = 2)**

| **Study (Year)** | Inclusion Criteria | Consecutive Inclusion | Consistent Reporting | Outcome Clearly Reported | Statistical Support | Quality Rating |
| --- | --- | --- | --- | --- | --- | --- |
| Fournier et al. (2020) ^21^ | Yes | Yes | Yes | Yes | No | High |
| Heinig et al. (2019) ^22^ | Yes | No | Yes | Yes | No | Moderate |

**Supplementary Table 3. Newcastle-Ottawa Scale for Retrospective Cohort Study (n = 1)**

| **Study (Year)** | Selection (max 4) | Comparability (max 2) | Outcome (max 3) | Total Score (max 9) | Quality Rating | Comments |
| --- | --- | --- | --- | --- | --- | --- |
| Tirado-Sánchez et al. (2021) ^23^ | 3 | 1 | 1 | 5 | Moderate | No control group; limited outcome depth |
